# Are we there yet? The road to faster and more efficient CAR T cell manufacturing

**DOI:** 10.1186/s13036-026-00666-5

**Published:** 2026-03-20

**Authors:** Leonor N. Matos, Margarida S. Costa, Margarida Serra, Marta H. G. Costa

**Affiliations:** 1 iBET - Instituto de Biologia Experimental e Tecnológica, Apartado 12, Oeiras, 2780-901 Portugal; 2 Instituto de Tecnologia Química e Biológica António Xavier, Universidade Nova de Lisboa, Av. da República, Oeiras, 2780-157 Portugal

**Keywords:** CAR T cell, Stem-like CAR T cells, Rapid manufacturing, Bioprocess parameters, Point‑of‑care manufacturing, Bioprocess control

## Abstract

Chimeric antigen receptor (CAR) T cell therapies have achieved clinical success in autologous treatment of hematological malignancies. However, their broader application remains limited. Beyond the biological challenges associated with the immunosuppressive microenvironment of solid tumors and the graft-versus-host disease risks inherent to allogeneic settings, the widespread adoption of CAR T cells is hindered by the high costs, long and complex vein-to-vein timelines and variability in product quality. Rapid CAR T cell manufacturing has emerged as an alternative paradigm that prioritizes shortened production workflows and preservation of naïve and stem-like T cell phenotypes with superior in vivo expansion, persistence and anti-tumor efficacy. This review examines rapid CAR T cell manufacturing from a bioprocess engineering perspective, focusing on how critical process parameters can be explored to shape CAR T cell phenotype and function within shortened timelines. We highlight key technological enablers, including automation, microfluidic systems, process analytical technologies, artificial intelligence-driven bioprocess control, quality control methodologies as well as safety considerations unique to accelerated workflows. Emerging CAR T cell manufacturing models, such as point-of-care production and in vivo CAR T cells generation, are also discussed. These insights outline engineering strategies to enable faster, more consistent and clinically effective CAR T cells.

## Introduction

Autologous CAR T cell therapies have achieved remarkable success in the treatment of hematological malignancies. However, the high costs (typically, 300–500 k € in Europe and the USA [1, 2]) and long vein-to-vein time (3–6 weeks [3–5]) are constraints to broader patient access. The long time between patient apheresis and drug product administration can constitute a significant hurdle for patients with aggressive tumors whose rapid disease progression can result in patient death before autologous cell therapies are ready for infusion. These constraints are exacerbated by the fact that patients are usually heavily pre-treated before they are considered eligible for CAR T cell therapy, resulting in T cell starting material with a more exhausted and terminally effector phenotype [6], compromising the potential efficacy of the final CAR T cell product.

Rapid CAR T cell manufacturing processes aim to address these limitations, shortening production timelines in autologous settings while preserving CAR T cells with a more naïve or stem-like phenotype. Such phenotypes are associated with improved clinical responses due to their superior in vivo expansion and persistence [7]. Contrary to conventional CAR T cell manufacturing workflows, which typically aim to generate large cell numbers (approximately 10⁸ cells) through prolonged ex vivo expansion, rapid manufacturing approaches rely on much lower cell doses (10^6^-10^7^ cells) [8–10], taking advantage of the presence of stem cell memory CAR T cells, that have higher in vivo proliferative activity [10].

Beyond autologous applications, the principles underlying rapid manufacturing may also benefit allogeneic and “off-the-shelf” CAR T cell therapies as low CAR T cell doses with high potency could show increased benefits to the patient and contribute to reduce cost of goods (CoGs), particularly when comparing with conventional manufacturing workflows that depend on higher doses but with higher proportion of less persistent effector T cells.

Although the success of CAR T cell therapies could be determined by biological factors such as the quality and composition of the starting T cell population, including their metabolic and differentiation state (often impacted by patient characteristics, disease state and pre-treatment regimen), bioengineering strategies can be applied to enhance product consistency and potency even in short manufacturing workflows.

In this review, we provide a comprehensive overview of rapid CAR T cell manufacturing strategies, focusing on biological determinants of product quality and critical process parameters that can be modulated during ex vivo culture. Additionally, we discuss technological enablers that could accelerate the adoption of rapid manufacturing workflows to generate clinically relevant CAR T cell products, as well as challenges associated with quality control (QC) release and safety. Point-of-care (PoC) and in vivo CAR T cell manufacturing models are also examined. We highlight how optimized and accelerated workflows can balance the need for speed with the use of minimal doses of CAR T cells while preserving stem-like T cell characteristics, enabling more accessible, affordable, potent and safer CAR T cell therapies.

## Overview of conventional vs. rapid CAR T cell manufacturing

Conventional CAR T cell manufacturing typically follows a multi-step workflow including leukapheresis, T cell enrichment, activation, genetic modification, ex vivo expansion, harvest and cryopreservation. These processes usually require 10–21 days of manufacturing (usually comprising a ˃7-day expansion step), resulting in a vein-to-vein time of several weeks when logistics (e.g., shipment from and back to the clinical site) and QC release testing are included.

**Fig. 1:**
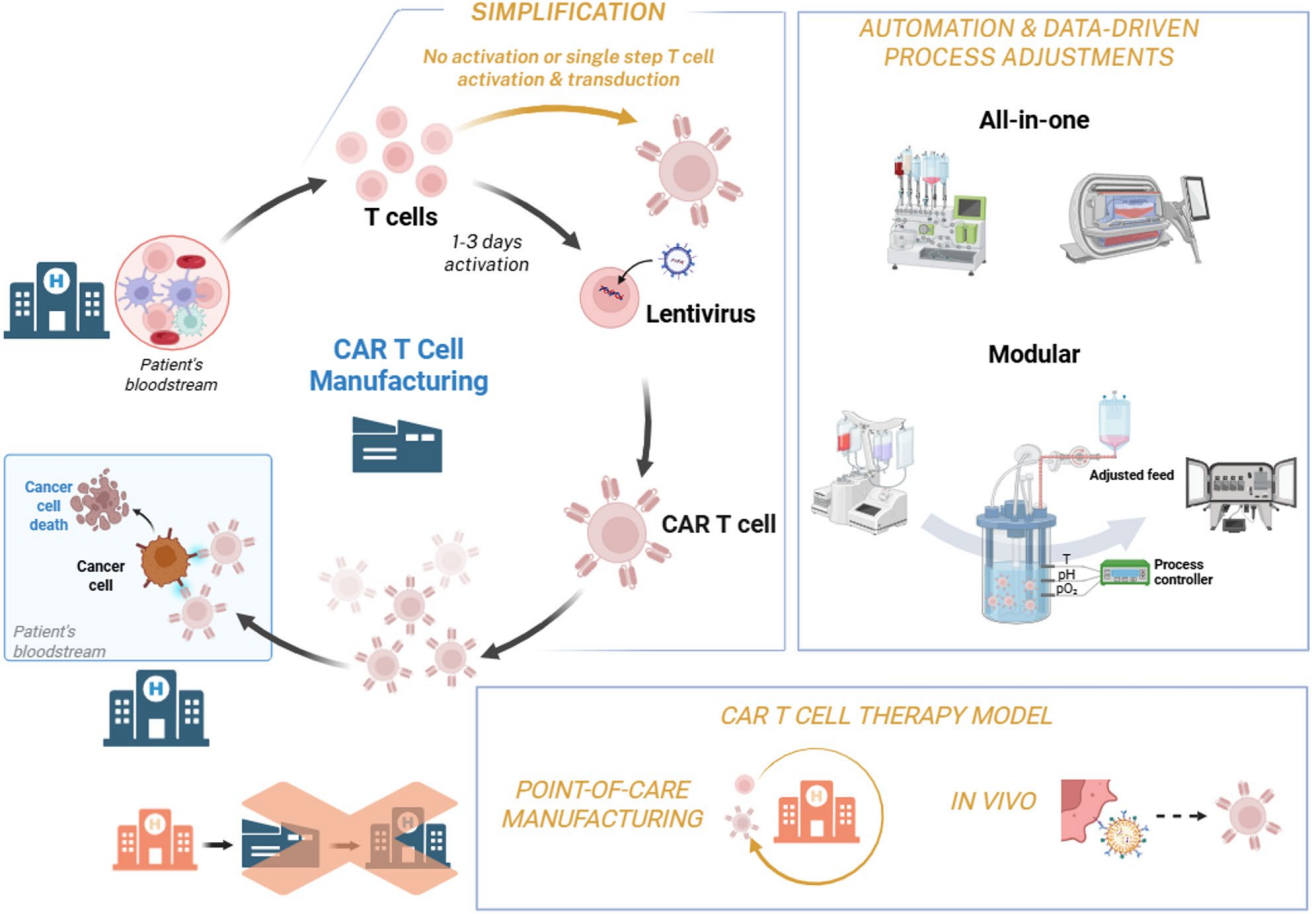
Schematic overview of conventional CAR T cell manufacturing workflow and key approaches to shorten production timelines. Process simplification can be achieved by shortening, combining or even eliminating manufacturing steps, such as shortening or bypassing T cell activation or through simultaneous T cell activation and transduction to generate CAR T cells. Automation and data-driven process adjustments leverage closed, all-in-one or modular manufacturing platforms, equipped with integrated sensors to enable real-time monitoring and adaptive control of critical parameters, accelerating production of CAR T cells with optimal phenotype while facilitating integration of distinct manufacturing steps. Alternative CAR T cell therapy models, including point-of-care manufacturing and in vivo CAR T cell generation, further reduce logistical delays associated with centralized production and product shipment

Rapid CAR T cell manufacturing strategies have emerged to directly address the limitations inherent to standard workflows. Rather than maximizing ex vivo expansion, rapid approaches prioritize phenotypic preservation of naïve/stem-like T cells, time-to-infusion and process simplification. Strategies focusing on either process acceleration (through PoC *or in vivo* CAR T cell manufacturing) or simplification of workflows (for instance, by eliminating T cell selection or activation steps) have been explored and might be supported by closed and automated platforms, reducing operator intervention and contamination risk (Fig. 1). Devices such as the Lonza Cocoon^®^, CliniMACS Prodigy^®^ and MARS Atlas enable integrated processing, from leukocyte input to final product [11]. Contributing to the advancement of rapid CAR T cell manufacturing workflows are also non-integrating gene delivery strategies, relying on mRNA-based CAR expression that is often supported by targeted lipid nanoparticle (LNP) delivery and have been explored both ex vivo and in vivo [12–15].

**Fig. 2:**
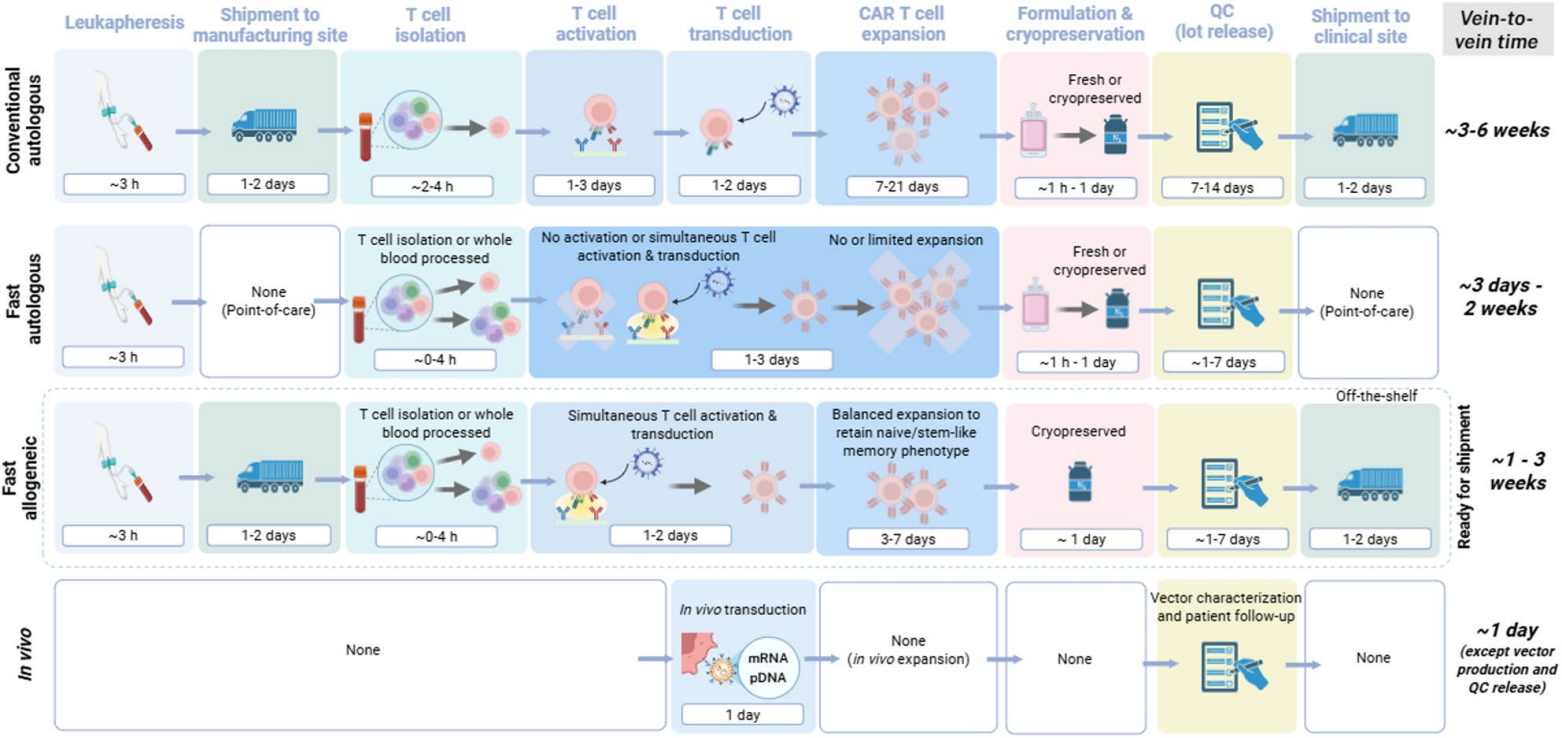
Comparison of conventional and rapid CAR T cell manufacturing workflows. Schematic overview of CAR T cell manufacturing strategies, highlighting key processing steps, timelines and vein-to-vein durations for conventional and rapid autologous CAR T cell manufacturing workflows, rapid allogeneic CAR T cell manufacture and in vivo CAR T cell generation approaches. Conventional autologous workflows involve centralized manufacturing, sequential T cell isolation, activation, transduction, prolonged ex vivo expansion, cryopreservation and extensive QC, resulting in vein-to-vein times of approximately 3–6 weeks. Fast autologous workflows compress or eliminate several steps, such as shipping, T cell activation and expansion, often enabled by point-of-care manufacturing, yielding vein-to-vein times as short as a few days. Rapid allogeneic strategies similarly shorten processing timelines while incorporating controlled expansion to preserve naïve or stem-like memory CAR T cell phenotypes. In vivo CAR T cell manufacturing represents a paradigm shift, relying on direct delivery of CAR-encoding nucleic acids (e.g., mRNA or DNA) to T cells within the patient, therefore eliminating ex vivo processing and reducing timelines to approximately one day, excluding vector production and QC. Approximate durations for individual steps are indicated to emphasize key contributors to the overall manufacturing time. QC – Quality Control

Rapid manufacturing approaches have shortened CAR T cell manufacturing workflows from 2 to 3 weeks (including vein-to-vein time) to as few days as solely 1 day [16], although QC release testing can still extend the overall vein-to-vein timeline to more than one week (Fig. 2). This is particularly relevant as it is estimated that approximately 30% of CAR T cell therapies are not infused in prescribed patients largely due to deterioration of their medical condition during the long period required to manufacture and release the CAR T cell product [17]. This clinical reality has motivated the scientific community to minimize delays between leukapheresis and treatment. In early 2024, Kite Pharma announced that the Yescarta manufacturing protocol had been shortened by 2 days without impacting product quality, illustrating that even incremental process acceleration within established workflows is possible [18].

Beyond process acceleration, other strategies have pursued workflow simplification, focusing on maximizing the proportion of naïve/stem cell memory and central memory CAR T cells in the final product at the expense of cell expansion, leveraging their superior in vivo proliferative activity. Indeed, while standard CAR T cell manufacturing requires extensive CAR T cell proliferation to reach target cell doses (typically 10⁸ cells), and, usually, involve ˃ 7 days of ex vivo expansion, which can result in enrichment of terminally differentiated effector subsets, rapid manufacturing intentionally limits CAR T cell expansion to ≤ 3 days to avoid their terminal differentiation and exhaustion. Of notice, these culture duration thresholds will be used throughout this Review to refer to conventional (˃7 days) and rapid (≤ 3 days) ex vivo expansion workflows. Despite efforts to accelerate CAR T cell manufacturing, autologous therapies are inherently constrained by their dependence on patient-derived cells. Therefore, when referring to ex vivo cell culture procedures, the culture duration thresholds defined here refer only to ex vivo processing time and do not include vein-to-vein time, which, even in accelerated workflows, typically extends beyond one week unless PoC strategies are implemented.

Clinical data supports the paradigm shift towards rapid CAR T cell manufacturing approaches. Products generated using rapid platforms, such as BMS-986,354 produced on the NEX-T platform, YTB323 manufactured via T-Charge™, FasTCAR™ or ultra-fast (UF)-CAR-derived products (i.e., UF-Kure19), have shown potent clinical efficacy despite being administered at doses 10-100-fold lower than their conventionally manufactured counterparts (Table 1). Importantly, differences in clinical performance are driven by the phenotypic characteristics of the generated CAR T cells rather than by the receptor design as supported, for instance, by the phase I clinical trial NCT04394650, where Bristol Meyer Squibb (BMS) NEX-T B-cell maturation antigen (BCMA)-targeting CAR T cell product BMS-986,354 was compared with orvacabtagene autoleucel, containing both the same CAR construct. Exploring significantly lower cell doses (20–80 × 10^6^ vs. 50–600 × 10^6^ CAR T cells), BMS-986,354 evidenced a higher proliferative capacity and potency [19]. Similarly, YTB323 CD19 CAR T cells, produced on T-Charge™ rapid manufacturing platform within 2 days, evidenced an overall response rate of 73% [20] compared to 52% achieved in the JULIET study [21] where tisagenlecleucel, sharing the same CAR transgene, was evaluated and administered at 10–100 times lower doses.

**Table 1:** Summary of preclinical studies and clinical trials employing accelerated CAR T cell manufacturing workflows

CAR T cell product (target); Disease; Company/Academic center	Manufacturing time	Vein-to-vein time	Platform/ manufacturing strategy	Activation strategy	Genetic engineering strategy/ timepoint	Cell numbers reached	Product characteristics	Cryopreserved	Doses	Outcomes	Ref.
Preclinical studies											
Ingenui-T (CD19) B-cell-driven autoimmune diseases Kyverna Therapeutics	˂3 days	NA	Centralized (Ingenui-T) From whole blood	Yes Dynabeads	Lentiviral transduction	38.5 ± 6.6 × 10^6^ cell (from 100 mL of whole blood) (0.68 ± 0.09-fold change)	CAR^+^ T cells (%): 45.1–54.5 Phenotype: . 93.9% T cell purity (CD3) . Effector/memory composition: 69.4 ± 4.8% in CD4^+^ and 46.6 ± 5.6% in CD8^+^ T cells	Yes	NA	In vitro killing: Robust CD19 specific cytotoxicity and serial rechallenge killing, achieving sustained target clearance at lower E: T ratios than conventional CAR T cells	[22]
CAR T cells (CD19) Leukemic cells ThermoFisher Scientific	1 day	NA	Centralized (G-Rex bioreactor) From frozen Peripheral Blood Cells (Leukopak)	Yes Dynabeads	~ 20 h lentiviral transduction	520 × 10^6^ cell (0.8-fold change)	CAR^+^ T cells (%): 76.4 Phenotype: . >95% T cell purity (CD3) . Higher Tn proportion and less exhausted phenotype, compared to conventional	Yes	NA	*In* vitro killing: Robust in vitro killing of CD19^+^ target cells, cytotoxic activity of the 24-hour CAR T cells trended higher than the 7-day CAR T cells after three days of ex vivo culture post-thaw	[23]
Non-activated CAR T cells (CD19) Leukemic cells University of Pennsylvania	1 day	NA	PoC (Static platform) From peripheral blood	None	20 h lentiviral transduction	NA	CAR^+^ T cells (%): 6–16	No	Non-activated: 2–20 × 10^5^ CAR T cell/mouse vs. Conventional: 3 × 10^6^ cell/mouse	In vivo killing: Tumor clearance at day 11 for highest dose and day 18 for lowest dose. Disease control for the > 90 days of the experiment for all mice receiving the highest dose and most mice receiving the lowest dose, outperforming conventional CAR T cells which led to relapse of all mice on day 17	[16]
MARS Atlas (CD19) Applied Cells	1 day	NA	PoC (MARS Atlas) From whole blood	Yes	Lentiviral transduction	20 × 10^6^ cells (from 30 mL of blood)	CAR^+^ T cells (%): 60–70 Phenotype: . >94% T cell purity. Twice more Tn population, compared to conventional	Both cyopreserved and ready for injection	NA	In vitro killing: CAR T cells showed killing at day 7, using repeated stimulation with target cells	[24]
DASH CAR T (CD19) Leukemic cancer cell lines Hrain Biotechnology	2–3 days	NA	Centralized (Static platform) From PBMCs	Yes Dynabeads	Retroviral transduction	NA	CAR^+^ T cells (%): . 48 h DASH CAR T: <20 . 72 h DASH CAR T: ~35–65 Phenotype: . Increased proportion of Tn (≥ 40% vs. ≤ 15% conventional CAR T) Function: . Higher secretion levels of IL-2, IFN-γ, TNF-α and G-CSF (> 1000 pg/mL vs. < 250 pg/mL conventional CAR T) . Higher expansion capacity post-thawing (after 3 days, > 4-fold expansion vs. < 1-fold expansion of conventional CAR T)	Yes	DASH: . 0.25, 0.5 and 1 × 10^6^ CAR^+^ T cells/mouse vs. Conventional: . 1 and 5 × 10^6^ CAR^+^ T cells/mouse	In vivo killing: Superior in vivo efficacy and overall survival benefit compared with conventional CAR T	[25]
DASH CAR T (CD276) Pancreatic cancer cell lines Hrain Biotechnology			Centralized (Static platform; RetroNectin-precoated plates to promote T cell transduction) From PBMCs				CAR^+^ T cells (%): . 48 h DASH CAR T: 5.52 . 72 h DASH CAR T: 59.3 Phenotype: . Increased proportion of Tn Function: . Higher expansion capacity post-thawing in co-culture with CD276^+^ target cells (after 7 days, > 10-fold expansion vs. < 5-fold expansion of non-transduced T cells)		DASH: . 1, 3 and 10 × 10^6^ CAR^+^ T cells/mouse vs. Conventional: . 1, 3 and 10 × 10^6^ CAR^+^ T cells/mouse	In vitro and in vivo killing: Higher anti-tumoral capacity in comparison to conventionally manufactured CAR T cells	[26]
Clinical trials											
huCART19-IL18 (CD19) NHL, CLL, ALL University of Pennsylvania	3 days	> 2 months	Centralized	Yes Dynabeads	Lentiviral transduction	NA	Phenotype: . Express IL-18 to directly stimulate IFN-γ production . >70% viability . Higher proportion of Tn and Tcm (> 70% vs. < 50% prior conventional anti-CD19 CAR T)	Yes	NCT04684563; administered doses of 3-300 × 10^6^ cells	ORR: 81% and CRR: 52%, evaluated at 3 months in patients with CD19^+^ lymphoma who are R/R to prior CD19 CAR T cell treatment CRS: 62% ICANS: 14%	[27]
GLPG5101 (CD19) NHL CellPoint, Galapagos	5 days	7–21 days	PoC	Yes	Viral	NA	Phenotype: Increased Tscm phenotypes compared with starting material Function: Robust in vivo expansion and long-term persistence for up to 21 months	No	NCT06561425 Phase I/II 35–250 × 10^6^ CAR^+^ viable T cells	ORR: 88% CRR: 83% Low rates of Grade ≥ 3 CRS (1.6%) and ICANS (1.6%)	[28–30]
GLPG5201 (CD19) CLL/RT CellPoint, Galapagos									Phase I/II 35–100 × 10^6^ CAR^+^ viable T cell	ORR: . at 35 × 10^6^ cell dose: 83% . at 100 × 10^6^ cell dose: 100% CRR: . at 35 × 10^6^ cell dose: 67% . at 100 × 10^6^ cell dose: 83% No CRS Grade ≥ 3 or ICANS	[31, 32]
PRGN-3005 UltraCAR T (MUC16) Ovarian cancer Precigen	1 day	1 day (with or without lymphodepletion)	UltraPorator/ PoC	None	Electroporation (non-viral transposon: Sleeping Beauty)	NA	Phenotype: Express membrane-bound IL-15 (mbIL15) for enhanced in vivo expansion and kill switch genes Function: In vivo persistence for up to 9 months	No	Phase I/Ib (NCT03907527) Without lymphodepletion: 0.1–65.5 × 10^5^/kg With lymphodepletion: 0.1–39 × 10^5^/kg	PRGN‑3005 was well tolerated up to at least 65.5 × 10^5^/kg with no dose‑limiting toxicities; 20% patients responded in at least one lesion	[33]
PRGN-3006 UltraCAR T (CD33) AML MDS CMML Precigen									Phase I/Ib (NCT03927261) Without lymphodepletion (C1): 1.8–50 × 10^5^ CAR T cells With lymphodepletion (C2): 4.4–83 × 10^5^ CAR T cells	PRGN-3006 infusion at up to 1 × 10^6^ cells/kg was well tolerated ORR: 30% in AML patients. No response in CMML or MDS patients	[34]
FasT CAR T (CD19) R/R ALL Gracell	2 days	14 days	Centralized	Yes Dynabeads	Lentiviral transduction	NA	CAR^+^ T cells (%): 35.4 Phenotype: . Tscm: 23.3% . Tcm: 36.1% . Tem: 33.2% Function: Superior in vivo proliferation and persistence of FasT-CAR T compared to conventional CAR T cells	Yes	Phase I (NCT03825718) 3.0-15.6 × 10^4^ CAR T cell/kg	CRR: 100% in 14 days Grade ≥ 3 adverse events occurred in 100% of patients CRS: 96% No CRS Grade ≥ 4 ICANS: 28% (Grade ≥ 3)	[10]
GC012F FasTCAR-T (CD19/BCMA), R/R NHL AstraZeneca Gracell	1 day	NA	Centralized	Yes Anti-CD3/CD28	Lentiviral transduction	NA	Phenotype: Dual-target CAR (CD19 & BCMA) Function: Robust in vivo expansion in all patients	NA	Phase Ib 3.7–30 × 10^4^ cells/kg	ORR: 100% (3 months); CRR: 77.8% (3 months); 62.5% (9 months)	[35, 36]
GC012F FasTCAR-T (CD19/BCMA), High-risk NDMM AstraZeneca Gracell									Phase I (NCT04935580) 1–3 × 10^5^ cells/kg	Median follow-up of 13.6 months: CRR: 95.5% Low grade CRS: 27% No CRS Grade ≥ 3 or ICANS	[36, 37]
UF-Kure19 UF-CAR (CD19) R/R NHL UHCMC	˂1 day	NA	PoC	Yes	Lentiviral transduction	NA	CAR^+^ T cells (%): 41 Phenotype: . Tn, Tcm, Tem and late Teff percentages consistent with pre-apheresis levels . 93% CD8^+^ T cells on day 10 Function: Robust expansion in vivo	Yes	Phase I (NCT05400109) 17.5 × 10^6^ CAR T cells	CRR: 80% (at 6 months); CRS: 30%	[38–40]
YTB323 T-Charge™ (CD19) R/R DLBCL Novartis	˂2 days	10 days	Centralized	Yes	Lentiviral transduction	NA	Phenotype: Retains the Tn and Tscm content of the input leukapheresis material Function: Robust expansion in vivo	Yes	Phase II (NCT03960840112.5 × 10^6^ CAR T cells	ORR: 88% CRR: 55% (3 months); 57% (6 months); 47% (12 months)	[20, 41, 42]
PHE885 T-Charge™ (BCMA) R/R MM Novartis	< 2 days	16 days	Centralized	Yes	Lentiviral transduction	NA	Phenotype: T cells with early memory phenotype were preserved in the final product Function: Robust expansion in vivo and long-term polyclonal persistence for > 6 months in 48% of patients	Yes	Phase I (NCT04318327) 2.5–20 × 10^6^ CAR T cells	ORR: 98%; 100% (at > 5 × 10^6^ doses) CRR: 42% (at 10 × 10^6^ CAR T cell doses)	[43–45]
CC-98,633/ BMS-986,354 NEX-T Platform (BCMA) R/R MM Bristol Myer Squibb	5–6 days	~ 45 days	Centralized	Yes (in cytokine-enriched culture media)	Lentiviral transduction	NA	Phenotype: Composed primarily of Tn and Tcm CAR^+^ cells with fewer effector and terminally differentiated CAR T cells Function: Robust in vivo expansion and persistence > 6 months	Yes	Phase I (NCT04394650) 20–80 × 10^6^ CAR^+^ T cells	ORR: 95% CRR: 46% CRS: 82% (median duration of 4 days) ICANS: 8% (median duration of 3 days)	[19, 46]
KITE-753 (CD19/CD20) R/R B-cell Lymphoma Kite Pharma, Gilead	5 days	NA	Centralized	Yes	Lentiviral transduction	NA	Phenotype: Dual-target CAR (CD19 & CD20) manufacturing process preserves Tn and Tscm cell populations	Yes	Phase I (NCT04989803) 3–20 × 10^4^ CAR T cells/kg	ORR: . at 3–10 × 10^4^ CAR T cells/kg: 64% CRR: . at 3–10 × 10^4^ CAR T cells/kg: 45% . at 20 × 10^4^ CAR T cells/kg: 100% Grade ≥ 3 adverse events occurred in 79% of patients, primarily cytopenias CRS Grade ≤ 3: 29% (median duration of 6.5 days) No CRS Grade ≥ 4 Low Grade ICANS: 14% (median duration of 6.5 days) No ICANS Grade ≥ 3	[47, 48]

The manufacturing time, vein-to-vein duration, platform and processing strategy (including T cell activation and genetic engineering approaches as well as whether the final product was cryopreserved) are indicated. Where available, achieved cell yields, administered doses and clinical outcomes following treatment with rapidly manufactured CAR T cells are summarized. Data are presented to maximize consistency across studies. However, differences in study design, populations and reporting limit direct cross-trial comparisons. When numerical values were not available, approximate estimations were derived from graphical data presented in the respective studies. NA indicates data not available. ALL – Acute Lymphoblastic Leukemia; AML – Acute Myeloid Leukemia; CLL – Chronic Lymphocytic Leukemia; CMML – Chronic Myelomonocytic Leukemia; CRR - Complete Response Rate; DLBCL – Diffuse Large B-Cell Lymphoma; MDS – Myelodysplastic Syndrome; MM – Multiple Myeloma; NDMM – Newly-Diagnosed Multiple Myeloma; NHL – Non-Hodgkin’s Lymphoma; ORR - Overall Response Rates; PoC – Point-of-Care; R/R – refractory/relapsed; RT – Richter’s Transformation; Tcm – central memory T cells; Teff – effector T cells; Tem – effector memory T cells; Tn – naïve T cells; Tscm – stem cell-like memory T cells. Conventional CAR T cells – ≥7-day manufacturing process

Overall, minimal CAR T cell expansion has shown success in treating cancer patients in autologous settings since stem-like CAR T cell phenotypes are preserved. FasTCAR™ (Gracell) enables next-day CAR T cell production and has shown encouraging clinical activity in B-cell acute lymphoblastic leukemia (B-ALL), with rapid expansion and manageable toxicity profiles [10]. Similarly, the UF-CAR platform enables CAR T cell generation in less than 24 h and early clinical results from UF-Kure19 in relapsed/refractory non-Hodgkin lymphoma showed encouraging results despite minimal ex vivo manipulation [38, 39].

## Biological determinants of rapid CAR T cell manufacturing success

Patient- and T cell-intrinsic features can dictate the success of rapid CAR T cell manufacturing, imposing biological constraints on what can be achieved within shortened ex vivo culture timeframes and influencing subsequent CAR T cell in vivo expansion, persistence and toxicity. CAR T cell efficacy has been linked to multiple T cell-intrinsic attributes, including T cell memory subset composition, exhaustion, CD4^+^/CD8^+^ T cell ratio, metabolic fitness, presence of regulatory T cells and cellular senescence [49].

### T cell subsets and differentiation state

The quality and effectiveness of CAR T cell products is tightly linked to their phenotype, as T cells move from naïve and stem-like memory states to highly differentiated effector, exhausted and senescent phenotypes, each carrying distinct implications for proliferative fitness, persistence and antitumor potency.

Briefly, naïve T cells are antigen-inexperienced T cells with highly proliferative potential and multipotency. Upon activation, naïve T cells give rise to stem cell-like memory T cells, which retain self-renewal capacity and the ability to differentiate into memory and effector subsets. Central memory T cells represent a more differentiated but still highly proliferative subset with long-term persistence, whilst effector memory T cells and terminal effector T cells are progressively differentiated towards immediate cytotoxic response and inflammatory cytokine production capacities, despite losing self-renewal and proliferative abilities.

Although effector T cells were initially considered as the target population for CAR T cell therapy due to their potent cytotoxicity, research shows that CAR T cell products enriched for early memory subsets achieve better disease control through higher expansion, durable anti-tumor response and prolonged in vivo persistence [50–55]. Consistently, central memory T cells have a greater capacity than effector memory T cells to persist in vivo and to mediate protective immunity, given their increased proliferative potential and stability in the absence of a recognized antigen [56, 57], whereas effector memory T cells show higher expression of genes associated with effector function and can mediate rapid tumor clearance in mice, but with more limited longevity [58].

Across several studies, a higher proportion of stem cell-like memory or central memory phenotype in CAR T cell products correlates with improved therapeutic outcomes. Enrichment of CD8^+^ central memory T cell phenotype in infused products associates with enhanced in vivo CAR T cell expansion [59]. Stem cell-like memory T cells are considered a preferred phenotype for CAR T cell products because of their self-renewal capacity, ability to generate other T cell subsets and their increased ability to engraft [60]. Consistently, stem cell-like memory CAR T cells have been shown to exhibit enhanced proliferative capacity and increased cytokine secretion upon CAR stimulation, resulting in better control of established tumors and resistance to tumor rechallenge in preclinical studies [54].

Moreover, manufacturing CAR T cells from pre-selected naïve, stem cell-like memory or central memory T cells results in more sustained and enhanced anti-tumor activity. In these settings, better clinical outcomes correlate with the frequency of stem cell-like memory T cells in the infused product [52, 53].

Prolonged ex vivo culture progressively enriches culture for terminally differentiated T cells and promotes CAR T cell exhaustion, a dysfunctional state of T cells driven by a chronic antigen exposure in tumors, or persistent infection, leading to reduced effector function, low proliferative and cytokine-producing capacity, high rates of apoptosis, and sustained expression of inhibitory receptors including PD-1, TIM-3 and LAG-3 [61, 62]. In vivo, this exhausted phenotype directly limits CAR T cell antitumor responses [63], highlighting the benefit of rapid manufacturing protocols designed to shorten culture duration and prevent exhaustion.

Beyond T cell differentiation state, the CD4^+^/CD8^+^ ratio of the CAR T cell product is also crucial for CAR T cell fitness. CD4^+^ T cells provide essential helper functions and regulate immune responses, whereas CD8^+^ T cells are primarily responsible for direct cytotoxic killing of target cells. Although CD8^+^ T cells are characterized by their cytotoxic profile, co-culture with CD4^+^ T cells has proven essential for CD8^+^ T cell expansion and function [64].

Taken together, these data support a model in which CAR T cell products enriched for naïve, stem cell-like memory or central memory T cells, with a balanced CD4^+^/CD8^+^ ratio and minimal exhaustion or senescence, confer superior proliferative potential, persistence and antitumor activity. Rapid manufacturing processes naturally align with this goal because they minimize prolonged ex vivo culture that would otherwise drive differentiation toward more effector phenotypes and promote exhaustion.

### Quality of starting T cell material

In the context of autologous therapies, patient-intrinsic factors, including age, disease state, prior treatments and immune cell profile, critically impact T cell functionality, ultimately compromising the success of CAR T cell therapies [65].

The quality of the apheresis material is therefore a critical determinant of CAR T cell manufacturing success, reflecting both the collection efficiency and the biological fitness of the collected lymphocyte population. Donor age is particularly impactful, as ageing affects the capacity of lymph nodes to sustain naïve T cell generation, resulting in an accumulation of less proliferative cells and a preferential loss of CD8^+^ T cells [66], with consequently higher CD4^+^/CD8^+^ ratios. Consistent with this, apheresis material from older donors has been shown to yield CAR T cell products of reduced quantity and quality, characterized by lower expansion capacity and a more differentiated phenotype in patient-derived cells [67], displaying lower transduction efficiencies and reduced cytotoxicity [68].

Moreover, in addition to age-related effects, clinical parameters at the time of apheresis strongly influence manufacturing outcomes as elevated platelet counts at apheresis have been correlated with reduced lymphocyte collection efficiency (< 40%) in patients with B cell malignancies [69]. Conversely, low platelet counts and low CD4^+^/CD8^+^ ratios in peripheral blood have also been linked to poorer clinical responses [70–72]. Particularly a CD4^+^/CD8^+^ ratio in peripheral blood at apheresis below 1/3 has been identified as a risk factor for manufacturing failure [70], whilst higher CD4^+^/CD8^+^ ratios at infusion (> 1.12) were associated with an increased risk of treatment failure [73]. In the context of rapid CAR T cell manufacturing, these baseline CD4^+^/CD8^+^ T cell ratios become particularly relevant. In conventional workflows, extended ex vivo culture often leads to a progressive decrease in the CD4⁺/CD8⁺ T cell ratio [74], but shorter ex vivo processes offer limited opportunity to reshape the cellular composition prior to infusion. As a result, rapid manufacturing approaches may preserve elevated CD4⁺/CD8⁺ T cell ratios inherited from the apheresis material.

Significant immune dysregulation is a hallmark of advanced hematological malignancies. High tumor burden drives T cell exhaustion and is often accompanied by systemic inflammation, with elevated levels of IL-6 and TNF-α, and high levels of IFN signalling associated with reduced CAR T cell expansion and a lack of durable responses [75]. Regulatory T cells (T_regs_) and circulating suppressive myeloid cells also contribute to an immunosuppressive environment, which supports tumor cell survival and negatively correlates with patient outcomes [75, 76]. Studies have also demonstrated that rapid CAR T cell expansion is proportional to pretreatment tumor burden, being one of the crucial factors for durable CAR T cell therapy response [77].

While chemotherapy and other previous therapies are extremely important to control disease progression, they can also be a determinant of T cell fitness and CAR T cell manufacturing success, with several studies demonstrating the impact of these agents on apheresis product quality and clinical outcomes. The use of alkylating agents, like bendamustine and high-dose melphalan, exhibits a detrimental effect on the apheresis peripheral blood mononuclear cells (PBMC) material up to 6–9 months after the last dose, highlighting the long-lasting impact of these regimens on T cell biology [78]. Recent use of bendamustine prior to apheresis increases the risk of manufacturing failure and is associated with negative treatment outcomes [70, 79]. High-dose melphalan and autologous stem cell transplantation, a procedure in which a patient’s own hematopoietic stem cells are collected and reinfused after high-dose chemotherapy to reconstitute hematopoiesis, also correlate with high probability of manufacturing failure, due to limited T cell transduction and CAR T cell expansion related with the accumulation of exhausted and senescent T cells and loss of naïve T cells [80]. Concordantly, cyclophosphamide and cytarabine chemotherapy have also been associated with the depletion of early lineage T cells [50]. In this context, delaying leukapheresis by at least three months following particularly toxic therapies has emerged as an important strategy to help restore T cell fitness. However, this delay can also inevitably prolong the overall CAR T cell therapy timeline and can offset some of the advantages sought with rapid manufacturing approaches.

Overall, cumulative chemotherapy exposure and the use of toxic regimens, particularly with short washout periods, adversely affect T cell fitness and may reduce target antigen expression, ultimately limiting product quality and efficacy [50, 81]. In combination with patient age, systemic inflammation and disease burden, these factors impair T cell quality and manufacturing efficiency. Patient- and donor-intrinsic factors become even more critical when considering rapid manufacturing protocols, since shorter processes, with little to no ex vivo expansion, have a limited ability to rescue impaired T cell fitness. As such, careful timing of leukapheresis based on immune, disease and treatment-related markers, including T cell phenotype pre-apheresis and prior treatments, is essential to identify candidates most likely to benefit from rapid protocols and to maximize the probability of manufacturing success.

### Metabolic programming and fitness

Distinct T cell differentiation states are characterized by different metabolic states to meet their energy and function demands. Whilst naïve T cells depend mainly on oxidative phosphorylation (OXPHOS) and fatty acid oxidation (FAO), effector T cells rely mostly on aerobic glycolysis [82–85]. As such, long-living proliferating T cells are associated with OXPHOS, while T cells with short lifespan associate with highly glycolytic cells [84]. Memory T cells have similar metabolic traits to naïve T cells, relying on OXPHOS and FAO, but with enhanced mitochondrial spare respiratory capacity, to facilitate rapid activation upon antigen re-encounter [86]. Consistently, inhibition of glycolysis has been shown to enhance the formation of memory CD8^+^ T cells [84].

Upon T cell receptor (TCR) activation, naïve T cells shift from FAO and OXPHOS to glycolysis, leading to differentiation into either highly-glucose dependent effector cells or low-glucose dependent memory T cells [87–89]. Although OXPHOS is much more efficient at meeting ATP demands, aerobic glycolysis allows the generation of metabolic intermediates important for cell growth and proliferation and maintaining redox balance (NAD^+^/NADH) in the cell [90–92]. This metabolic choice, to not maximize ATP production but to support the biosynthetic and redox demands of cell division, is known as the Warburg Effect, characteristic of proliferating cells, as cancer and activated lymphocytes66. Moreover, it has been shown that aerobic glycolysis is essential for full effector function, with IFN-y production post transcriptionally controlled by glycolysis engagement and disengagement [87].

This metabolic reprogramming is accompanied by mitochondrial remodelling. In fact, mitochondria support activation, proliferation, differentiation and effector function of T cells, and undergo significant changes as T cells transition from naïve to effector and memory states [86, 93]. During this transition, mitochondrial morphology changes: mitochondria increase their mass and spare respiratory capacity and adopt structures that support either rapid effector function or long-lived memory [86]. In the context of chronic stimulation, however, mitochondria become dysfunctional, impairing respiration and leading to the accumulation of oxidative stress. This oxidative stress is sufficient to impair T cell proliferation and self-renewal, promoting terminal T cell differentiation and consequent exhaustion [82, 85, 93]. Conversely, enhanced glycolysis is characteristic of terminally exhausted T cells, due to their defective mitochondria [85].

CAR T cell persistence, resistance to exhaustion and antitumor activity are tightly linked to their metabolic profile. Products with robust mitochondrial function, adequate capacity for glycolysis on activation, and the ability to adopt an FAO/OXPHOS-rich memory program, are better suited to withstand brief ex vivo stimulation and then expand and persist in vivo, as in rapid CAR T cell manufacturing workflows. In this context, products enriched in metabolically fit, memory-like cells show better in vivo expansion and durability than highly glycolytic, terminal effector-like cells.

## Critical process parameters in rapid CAR T cell manufacturing

**Fig. 3:**
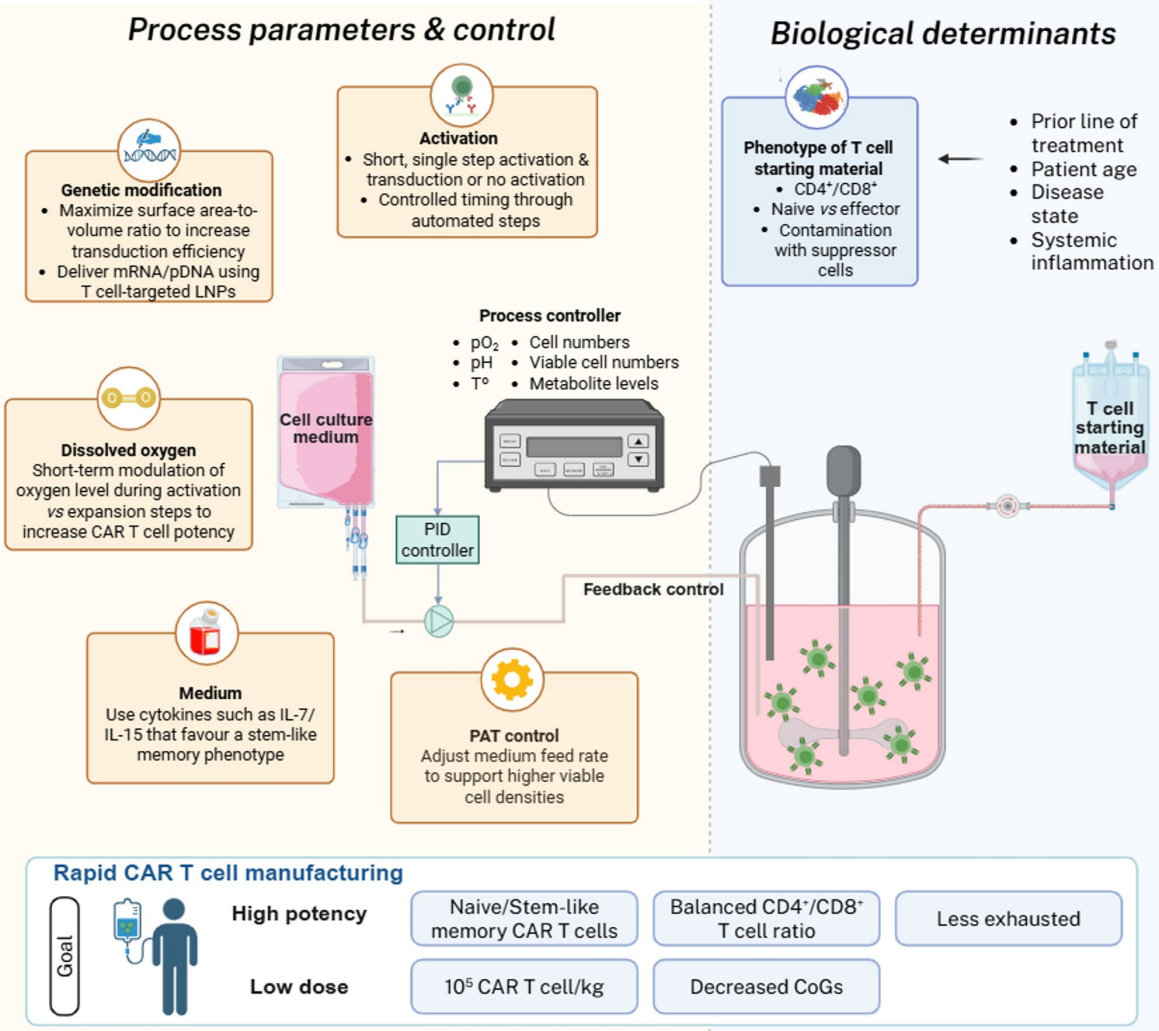
Bioprocess parameters and biological determinants in rapid CAR T cell manufacturing. Schematic representation of key process parameters, control strategies, and biological determinants that collectively shape CAR T cell quality in rapid manufacturing workflows. Critical bioprocess variables, including genetic modification strategy, T cell activation, impact of dissolved oxygen and culture medium formulation, are integrated with process control systems, such as feedback-regulated bioreactors, that are online monitored through PAT tools to regulate pH, pO₂, temperature, cell density and metabolite levels. Intrinsic biological determinants of the starting T cell material, including CD4^+^/CD8^+^ ratio, differentiation state (naïve vs. effector T cells) and contamination with suppressive cell populations, are influenced by patient-specific factors such as prior treatments and systemic inflammation. Together, these engineering and biological inputs define the achievable product attributes of rapid CAR T cell manufacturing. PAT - process analytical technologies; PID - proportional-integral-derivative

Besides the characteristics of the T cell starting material, which can influence clinical outcomes, specific T cell subpopulations can be enriched or their generation favored during manufacturing. Herein, we discuss how distinct process parameters can be modulated in rapid manufacturing processes to promote the generation of CAR T cells with increased potency and persistence (Fig. 3).

### Culture duration

Culture duration during CAR T cell manufacturing shapes both the differentiation and exhaustion state of the final product, as well as the CD4^+^/CD8^+^ T cell balance [49].

As CAR T cell culture is extended to maximize cell yields, a higher proportion of the cell product becomes terminally differentiated and naïve and stem memory T cells are replaced by effector T cells with lower long-term persistence in vivo and consequently reduced antitumor activity [94]. Additionally, prolonged CAR T cell expansion can lead to T cell exhaustion but also result in epigenetic changes with DNA hypermethylation, resulting in poor therapeutic potential [95].

Moreover, during ex vivo CAR T cell manufacturing, culture duration has a major influence on CD4^+^/CD8^+^ T cell dynamics, with longer expansion typically resulting in a progressive enrichment of CD8⁺ T cells due to their higher proliferative responsiveness to common activation and cytokine conditions [74]. As cultures are extended from a few days to 7–21 days, the CD4^+^/CD8^+^ T cell ratio often declines. In contrast, rapid manufacturing tends to preserve a CD4^+^/CD8^+^ T cell balance closer to baseline, thereby maintaining a more physiologic CD4^+^/CD8^+^ T cell mixture in the final product [23, 49].

CAR T cell rapid manufacturing platforms prioritize short culture maintaining a balanced CD4^+^/CD8^+^ T cell mixture enriched in less differentiated cells, deliberately trading maximal ex vivo fold expansion for a less exhausted cellular product with superior in vivo persistence [96, 97].

Noteworthy, even in allogeneic CAR T cell therapies, where maximizing cell yields is desirable to enable treatment of larger patient populations and reduce CoGs, dose escalation does not necessarily translate into improved clinical efficacy [98]. Instead, administering lower doses of highly potent CAR T cells may offer comparable or superior therapeutic benefit. Therefore, this supports the adoption of shorter manufacturing workflows in both allogeneic and autologous settings, where doses as low as 10^5^ cells/kg have shown promising clinical outcomes [99].

### T cell activation

 Ex vivo activation of T cells is conventionally performed using anti-CD3 and anti-CD28 antibodies that could be either attached to paramagnetic beads (Dynabeads™) (such as in tisagenlecleucel manufacture) or incorporated in a soluble and biodegradable nanomatrix (TransAct™). While Dynabeads require a subsequent step to be removed and additional QC is required to ensure no residual magnetic beads remain, TransAct™ can be removed with a washing step and recent studies have shown that this washing step could be skipped without negatively impacting T cell expansion or their killing capacity in vitro [100].

The duration [101, 102] and intensity [103] of T cell activation, as well as the choice of activation cues [67], critically influence CAR T cell differentiation and product quality. Short activation periods, such as 2 days of CD3/CD28 co-stimulation, promoted enrichment of memory stem T cells in comparison with longer activation stimulus (10 days) [101]. Conversely, prolonged or overly strong activation protocols have been associated with increased differentiation and reduced product quality [62]. Interestingly, other studies have shown that even one-day interventions introduced at critical moments of the CAR T cell activation workflow can contribute to determine T cell fate. Chen, et al. have identified that a transient resting period of one day (without medium supplementation with IL-2) after activation supports expansion of stem-like memory T cells and reduces exhaustion [104]. Importantly, controlled modulation of activation timing can be promoted in stirred-tank bioreactors, where the contact profile between T cells and anti-CD3/CD28 microbeads to trigger or stop activation, can be tailored through optimal agitation regimen [105].

Besides being a critical step to maximize CAR T cell yields in manufacturing workflows, activation is also essential to increase the efficacy of cell transduction (by facilitating uptake of viral particles) [106]. Indeed, T cell activation induces a transient upregulation of low-density lipoprotein receptor (LDL-R), which serves as the primary entry receptor for vesicular stomatitis virus G protein (VSV-G)-pseudotyped lentiviral vectors. The post-activation peak in LDL-R expression enhances viral binding and membrane fusion, effectively increasing transduction efficiency [107, 108].

Nonetheless, a recent study has demonstrated that non-activated T cells can be successfully transduced with lentiviral vectors. Although lentiviral gene transfer is generally less efficient in resting and non-activated T cells, Ghassemi and colleagues showed that transduction efficiency could be substantially improved by maximizing the surface area-to-volume ratio of the culture vessel and performing a transient serum starvation step. CAR T cells generated using this strategy exhibited higher anti-tumoral activity in in vivo models in comparison with activated CAR T cells [16]. Indeed, bypassing the activation step, which trigger T cell expansion and differentiation, can preserve a higher proportion of T cells with a more naïve phenotype that have higher persistence in vivo relatively to more differentiated T cells [55].

Other strategies using non-viral vectors can efficiently deliver CAR constructs into both resting and pre-activated T cells via electroporation. Transposon systems have been explored to transfect non-activated T cells, streamlining CAR T cell production [109].

Overall, although activation is a key determinant of CAR T cell phenotype, expansion and function, in the context of rapid manufacturing protocols, it can be shortened or even skipped, preserving a more naïve population with a highly proliferative potential in vivo while still allowing efficient genetic modification under optimized conditions.

### Vector exposure

Multiplicity of infection (MOI), defined as the number of viral vector particles added per T cell during transduction [110], represents a critical process parameter in CAR T cell manufacturing as it determines both the proportion of CAR^+^ cells and the vector copy number (VCN) per cell, which together shape product potency, persistence and safety.

Generally, increasing MOI enhances transduction efficiency, raising the proportion of T cells that express the CAR, which in turn increases the fraction of infused cells capable of specifically recognizing and engaging the target tumor antigen. A higher proportion of CAR‑expressing, antigen‑specific T cells can translate into stronger antitumor activity and improved clinical responses. Optimal synchronization of T cell activation-dependent LDL-R upregulation and vector exposure may reduce the need for excessively high MOI, therefore mitigating the risk of insertional mutagenesis and long-term genotoxicity associated with high VCN [110, 111]. Moreover, high CAR expression can promote tonic signalling, where target-independent T cell activation could lead to premature T cell exhaustion and impaired effector function [110, 112–114]. Consequently, regulatory agencies recommend keeping VCN within predefined limits, commonly around five copies per genome, to balance antitumor efficacy with long-term safety [115].

In the context of rapid manufacturing processes with limited ex vivo expansion, MOI becomes particularly important, as slightly higher values may be employed to rapidly achieve the desired proportion of CAR^+^ cells within a timeframe of hours to a few days. However, this strategy must be carefully balanced against acceptable VCN thresholds and the potential negative impact of high integration burdens on T cell fitness. Therefore, optimizing the MOI to achieve a defined CAR⁺ fraction using a VCN window that maximizes potency while maintaining an acceptable safety profile is a crucial step in process development for rapid CAR T cell manufacturing [25, 116].

### Culture density

Culture density is a key parameter in CAR T cell manufacturing as it influences proliferation kinetics, nutrient and cytokine gradients and, ultimately, cell viability and phenotype [117]. At excessively low densities, activated T cells may exhibit poor survival and expansion, reflecting a dependence on autocrine and paracrine signals that are insufficient when cells are too dispersed [118, 119]. On the other hand, very high cell densities without adequate oxygenation and nutrient supply lead to waste metabolite accumulation, local hypoxia and nutrient depletion, which can impair proliferation and bias cells toward more terminally differentiated and exhausted states unless counterbalanced by bioprocess strategies such as medium perfusion [117, 120, 121].

Experimental optimization studies indicate that intermediate seeding densities on the order of 10^5^ cell/mL represent an ideal range for ex vivo T cell expansion. Ghaffari, et al. showed that starting at approximately 2.5 × 10^5^ cell/mL yielded the highest fold expansion, whereas lower densities (≤ 1 × 10^5^ cells/mL) resulted in poor growth and higher densities (≥ 1 × 10^6^ cell/mL) progressively reduced expansion despite sufficient stimulation [119]. Consistently, Ma, et al. demonstrated that cultures initiated at very low density (1 × 10^4^ cell/mL) failed to expand and instead lost viable cells over time, confirming the existence of a critical density threshold below which proliferation cannot be sustained [118].

In rapid CAR T cell manufacturing, setting the seeding density high enough to promote cell-cell signaling but low enough to avoid mass transfer limitations is a relevant parameter to determine whether the product remains viable and enriched in less differentiated T cell subpopulations or transitions toward a metabolically stressed, less fit phenotype by the time of harvest. Although rapid manufacturing protocols are not primarily designed to maximize cell expansion yields, they can still leverage perfusion strategies to operate at very high cell densities, up to around 2 × 10^7^ cell/mL as demonstrated in recent studies, without compromising product quality [121, 122]. By enabling higher cell densities, perfusion allows manufacturing in smaller culture volumes while maintaining favorable CAR T cell phenotypic and functional attributes, which becomes particularly attractive for PoC manufacturing where minimizing equipment footprint and enabling parallel treatment of multiple patients are key operational constraints.

### Cytokine-driven CAR T cell differentiation and expansion

During CAR T cell manufacturing, cytokines play a crucial role by promoting T cell activation and proliferation as well as in the maintenance of less differentiated subpopulations that may enhance in vivo persistence and antitumor efficacy [7, 10, 93].

Cytokines such as IL-2, IL-7, IL-15 and IL-21 are often added to CAR T cell culture media, driving CAR T cell phenotype. Although IL-2 is an important cytokine, it may promote a more terminally differentiated CAR T cell phenotype that, long-term, is linked to decreased anti-tumor efficacy and persistence in vivo. In fact, by upregulating perforin, granzyme B, and IFN-γ and inhibiting memory cell markers like BCL6 and IL7RA, IL-2 promotes terminal effector T cell differentiation, potentially reducing the proportion of memory cells [123, 124].

In opposition, IL-7/IL-15 combination favors stem-like memory T cells with lower expression of exhaustion markers when compared to IL-2. Indeed, while CAR T cells expanded in medium supplemented with IL-2 show robust killing of tumor cells both in vitro and in vivo, overtime, CAR T cells expanded in IL-7/IL-15 have been reported to outperform IL-2-expanded CAR T cells, showing increased expansion and improved in vivo persistence [60, 117]. IL-21 supplementation further promotes memory phenotypes but may limit overall fold expansion, in comparison to IL-2 supplementation [115, 125].

In conclusion, CAR T cell manufacturing requires tailored cytokine supplementation, with most current protocols using combinations of IL-2, IL-7, IL-15 and IL-21. However, the optimal cytokine cocktail depends on the desired product profile, tumor biology and clinical context.

### Metabolic and phenotypic programming through oxygen control

Dissolved oxygen (DO) is a critical process parameter in CAR T cell manufacturing, impacting T cell metabolism, differentiation state, proliferation and functional fitness [126, 127]. Although the majority of the CAR T cell manufacturing protocols do not control the percentage of oxygen dissolved in the culture medium, in vivo, T cells reside in environments with oxygen tensions significantly lower than atmospheric levels [128].

Tuning the oxygen level can be used to metabolically prime CAR T cells while avoiding their terminal differentiation as oxygen availability directly regulates the balance between oxidative phosphorylation and glycolysis through oxygen-sensitive signalling pathways, including hypoxia-inducible factor (HIF) [129]. Cunha and colleagues have reported that short-term oxygen modulation is sufficient to reprogram T cell metabolic state and functional potential. Interestingly, the team showed that low oxygen levels (1% O_2_) during the activation phase of CD8^+^ T cells can increase their mitochondrial mass and enhance the anti-tumor function of CAR T cells [126]. This study highlights that oxygen control can act as a rapid and potent signal to determine cell fate and, in rapid manufacturing workflows, even short and transient exposure to distinct oxygen levels could be potentially explored to shape T cell fate and product quality. Interestingly, although some studies have reported that low oxygen levels can impair T cell proliferation [130, 131] as well as reduce transduction efficiency [130], T cells activated under hypoxic conditions have also shown increased protection from activation-induced cell death [132].

The relevance of DO control during CAR T cell manufacturing was further highlighted by Song, et al., who performed a systematic side-by-side comparison of CAR T cell expansion across multiple manufacturing platforms (CliniMACS Prodigy, Xuri rocking platform, G-Rex, static bag culture) with distinct oxygen transfer characteristics. Their study revealed that differences in oxygen availability, rather than the platform per se, were key determinants of cell expansion, metabolic state and CAR T cell phenotype [133].

Advanced culture systems, including stirred-tank bioreactors, wave bioreactors, hollow-fiber systems and microfluidic platforms, offer improved oxygen mass transfer and enable more precise DO control. Exploring these systems to work within DO ranges that preserve stem-like phenotypes while promoting optimal transduction levels can represent a useful tool towards the manufacture of more consistent CAR T cell products across distinct donors. Additionally, tightly controlled DO levels can limit excessive reactive oxygen species (ROS) accumulation and DNA damage [134, 135] during ex vivo CAR T cell culture while maintaining sufficient oxidative capacity to support cell expansion.

Importantly, oxygen consumption profiles may serve as early indicators of CAR T cell activation and fitness [136], supporting Process Analytical Technology (PAT)-enabled process control.

### Genetic engineering strategies

Genetic engineering remains a bottleneck in rapid CAR T cell manufacturing, as conventional viral transduction workflows typically require T cell activation and complex QC to ensure stable integration and safety, hindering fast processes. Viral (lentiviral and retroviral) transduction is still the most common method employed to generate CAR T cells, supporting stable transgene expression. From a bioprocess perspective, lentiviral vectors offer flexibility for rapid manufacturing, as they can transduce minimally activated or even resting T cells, whereas retroviruses require cell division for genomic integration. The ability of lentiviral vectors to deliver genetic cargo to quiescent T cells has been explored to shorten CAR T cell manufacturing timelines [16, 137]. Nonetheless, although all commercially available products are manufactured following lenti- or retro-viral transduction, typically resulting in ˂50% transduction efficiency, new modalities are emerging to generate CAR T cells. Those include CRISPR/Cas9 [138] or transposon [139] (such as Sleeping Beauty and piggyBac) approaches.

Transposon represent attractive alternatives to viral vectors for rapid CAR T cell manufacturing because they enable stable genomic integration and can be applied to modify minimally activated T cells [140] without the need for viral production. Sleeping Beauty-engineered CAR T cells have already shown anti-leukemic activity in clinical settings with acceptable safety profiles in early phase studies [141]. Remarkably, transposons have allowed successful introduction of the CAR gene in non-activated T cells [141], which can contribute to shorten manufacturing timelines. Nonetheless, transposon-based approaches typically rely on electroporation, which can transiently impair cell viability and functionality, an important consideration when cell numbers and culture time are limited. Furthermore, integration remains semi-random, necessitating careful monitoring of genomic integrity. Indeed, while transfection efficiencies above 50% have been reported [142], similarly to viral transduction methods, there are risks associated with insertional mutagenesis and CAR T cell lymphoma has been reported when piggyBac-modified CAR T cells were infused [143, 144], which underscores the importance of long-term monitoring.

CRISPR/Cas9-based genome editing offers a powerful approach for precise insertion of CAR constructs into defined genomic *loci*, mitigating risks associated with random viral integration [145]. Targeted integration can reduce variability in CAR expression, minimize tonic signalling and improve consistency across products [146]. These attributes are particularly attractive for rapid manufacturing, where process-induced and T cell donor heterogeneity is more challenging to correct downstream. However, editing efficiencies remain relatively low in T cells and might require enrichment steps that are incompatible with accelerated timelines.

Non-integrating gene delivery strategies, such as mRNA-based CAR expression, offer the fastest route to generate CAR T cells. Given the non-integrating nature of mRNA and the transient CAR expression, this is considered a safe approach. Recent advances in LNP technology have expanded the potential of mRNA delivery beyond electroporation, showing prolonged CAR T cell efficacy in vitro when compared with CAR-mRNA delivery through electroporation [12]. Further contributing to reduce cell culture time, Metzloff and colleagues developed activating LNPs functionalized with CD3 and CD28 antibody fragments, enabling one-step T cell activation and transfection to generate anti-CD19 CAR T cells [13]. Besides minimizing handling steps and shortening manufacturing timelines, this approach can also contribute to reduce CoGs as it eliminates the need for activation beads. Remarkably, LNP-mediated delivery of CAR-encoding mRNA has enabled the generation of CAR T cells directly in vivo [14]. However, transient expression inherently limits CAR persistence and may require repeated dosing or not be suitable for challenging diseases requiring long-term control.

## Technological enablers of rapid CAR T cell manufacturing

**Fig. 4:**
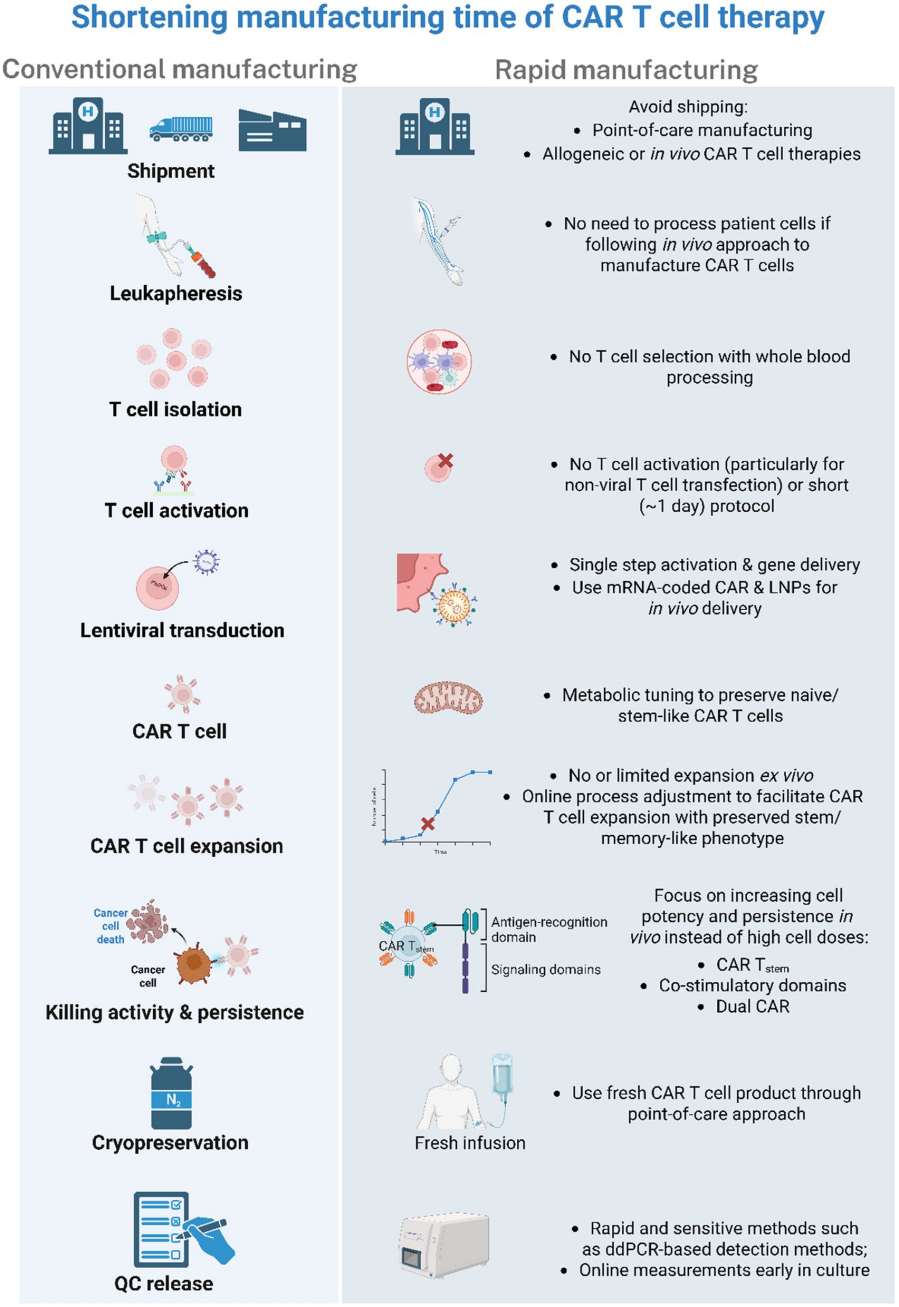
Strategies enabling rapid CAR T cell manufacturing across the production workflow. List of strategies enabling rapid manufacturing across distinct manufacturing steps. Approaches range from elimination of T cell selection through whole blood processing, use of non-viral gene delivery methods such as mRNA delivered by LNPs up to minimal or no ex vivo CAR T cell expansion to limit differentiation and exhaustion as well as faster methods supporting QC release

Rapid CAR T cell manufacturing workflows encompass not only the manufacturing of the drug product itself but also accessing and shipping the apheresis starting material, QC release testing and transportation to the clinical site prior to patient administration. Therefore, technological enablers of fast CAR T cell manufacturing processes should also consider CAR T cell models (i.e., PoC vs. centralized production) and the design of streamlined QC strategies compatible with shortened timelines. Indeed, strategies implemented across the entire CAR T cell manufacturing workflow, from handling of the starting material to genetic engineering and product release can be modulated individually or in combination to compress manufacturing timelines while preserving or even potentiating CAR T cell fitness (Fig. 4).

### Point-of-care manufacturing

While allogeneic CAR T cells obtained from healthy donors could enable off-the-shelf CAR T cell therapies, in autologous settings, reducing vein-to-vein time in a centralized manufacturing model is inherently constrained by the need to ship patient apheresis material to a remote facility where cells are manufactured and, after production, return the CAR T cell product to the treatment center.

PoC manufacturing avoids these logistical delays by using fresh starting material and eliminating the need for product cryopreservation and shipment. As a result, PoC approaches can substantially shorten the vein-to-vein interval. Platforms such as the Prodigy^®^ can operate within hospital environments, supporting rapid manufacturing workflows of autologous CAR T cell therapies while minimizing cross-contamination risks. Nonetheless, process standardization and reproducibility across decentralized sites represent significant hurdles as not only donor variability, but also operator expertise and site-specific infrastructure can introduce additional sources of variability, complicating process control. Additionally, QC assays required for product release in rapid PoC manufacturing workflows, are often centralized or rely on methods with long turnaround times and could therefore become the rate-limiting step in PoC models.

Additionally, it is important to note that lymphodepleting chemotherapy (frequently involving fludarabine and cyclophosphamide, administered alone or in combination) implemented prior to CAR T cell infusion still require several days [147] and, therefore, unless this step is removed, it remains a meaningful contributor to the overall vein-to-vein time even in PoC manufacturing models.

### Automation platforms and process analytical technology-driven bioprocess adjustments

Automated and closed platforms, such as bioreactors, can streamline manufacturing processes by shortening production timelines and minimizing process variability while requiring a smaller workforce. These platforms can reduce the need for high-grade cleanroom facilities, lowering costs and, importantly, in the context of short manufacturing processes, they can also reduce hands-on operator time. Several bioreactor configurations, including stirred-tank bioreactors [121, 148, 149], wave bioreactors [133] and hollow-fiber bioreactors [150], have been successfully applied to generate T cells/CAR T cells. The Prodigy^®^ (Miltenyi) and the Cocoon^®^ (Lonza) are amongst the most widely used platforms [133, 151, 152], particularly in PoC manufacturing [153], as their closed and semi-automated design enable a “one-device-per-patient” approach. However, integration of emerging technologies into such platforms remains challenging and systems like the Prodigy^®^ currently lack integrated process analytical technology (PAT) tools, which are essential to facilitate real-time monitoring and control of CAR T cell manufacturing processes. In contrast, modular manufacturing approaches offer greater flexibility and can support parallelized CAR T cell production for multiple patients while optimizing equipment utilization. By streamlining equipment usage, modular workflows can therefore contribute to reduce patient wait times associated with limited manufacturing capacity [11].

In the context of rapid manufacturing workflows, PAT tools allowing inline and online monitoring of product quality will likely assume a critical role over offline measurements, supporting process adjustments in real-time and aiding in a faster QC release while also minimizing risks of contamination and contributing towards automation.

Technologies such as Raman spectroscopy, UV spectroscopy, near-infrared and mid-infrared spectroscopy, capacitance probes or holographic microscopes [149, 154–160] integrated in the cell culture vessels can be used to estimate process parameters such as cell numbers and metabolites concentration, supporting, for instance, the identification of the harvest timepoint or medium change rates. The iLine F (Ovizio), for instance, can continuously estimate cell numbers without wasting limited cell samples given its closed-loop setup [154]. Similarly, capacitance probes can be used to quantify cell viability as viable cells, with an intact cell membrane, function as capacitors when exposed to an electric field [155, 161].

These tools, capturing bioprocesses in real-time, can generate large volumes of data. If coupled with Artificial Intelligence (AI) to interpret the data, this information can inform automated decision-making and enable integrated process adjustments, ultimately yielding CAR T cell products with greater clinical relevance, including enhanced potency and reduced manufacturing costs.

### AI enablers

The use of AI and machine learning (ML) can open new avenues to enable rapid manufacturing of CAR T cells with enhanced therapeutic potential, contributing to shortening development and manufacturing times, reducing CoGs and improving cell quality by facilitating data-informed decisions on process control.

Process optimization, for instance, can be driven by predictive models that support the identification of optimal cell seeding density, feeding strategy, media formulation, incubation times or genetic engineering procedures. For example, a ML pipeline has been applied to optimize T cell culture media formulations, maximizing expansion robustness across donors within relatively short (≤ 6-day) culture periods [162].

AI-enabled digital twins, allowing a virtual representation of on-going processes, offer a powerful framework to support real-time decision-making. Digital twins integrating online sensor data (e.g., oxygen consumption, lactate production, cell size dynamics, or capacitance measurements) can be used to predict CAR T cell expansion kinetics and viability trajectories. Egan and colleagues have shown that digital twins have the potential to predict CAR T cell concentration. This can be therefore used to predict the optimal harvest time point of CAR T cells, avoiding processes to extend beyond the minimum required to reach target cell doses [163]. In the rapid CAR T cell manufacturing paradigm, such predictive control strategies are particularly relevant, as even modest extensions in culture duration can compromise the desired naïve or stem-like T cell phenotype.

A critical opportunity for AI in rapid CAR T manufacturing lies in early prediction of product quality and potency. Recent studies have demonstrated that AI-driven analytical platforms integrating phenotypic, functional and metabolic features can predict T cell fitness from early in-process measurements [164]. ML models trained on early culture readouts, such as mitochondrial activity, metabolic signatures and surface marker expression, could potentially be used to predict in vivo antitumor efficacy and persistence. Applied to rapid workflows, such models could mitigate the need to wait for later-stage potency assays that are incompatible with accelerated timelines. Indeed, AI and ML approaches can significantly contribute to accelerated QC and release testing, which remains one of the most significant bottlenecks in rapid CAR T cell manufacturing. Approaches like the one proposed by Strutt, et al., who explored long read sequencing coupled with ML, can contribute to rapidly (˂24 h) identify low abundant microbial contaminants present in T cell cultures, shortening sterility tests for bacteria and fungi that currently take weeks [165]. Integration of multi-omics data (e.g., transcriptomics, metabolomics, epigenetic profiling) [166] could provide a AI-enabled opportunity to refine predictive models and inform surrogate markers of potency that are compatible with rapid product release.

Despite these opportunities, the need for model transparency and interpretability to meet regulatory requirements must be addressed before AI can be routinely deployed in rapid CAR T cell manufacturing.

### Microfluidic systems for high-density culture

Microfluidic culture systems have emerged as a promising technological enabler for rapid CAR T cell manufacturing as miniaturized culture compartments can tightly regulate mass transport, enhancing nutrient delivery, gas exchange, and waste removal. Another important advantage of microfluidic systems is their compatibility with integrated and real-time monitoring. Non-invasive modalities such as optical imaging, impedance-based sensing and profiling of biophysical signatures through cell trajectory modulation assay, for instance, can be incorporated into microfluidic devices, enabling continuous assessment of cell number, viability and functional activity [167, 168]. These capabilities could support closed-loop control strategies, aligning with emerging PAT- and AI-enabled frameworks for compressing decision-making and release timelines in rapid CAR T cell manufacturing. Recent work has demonstrated that automated and closed 2 mL microfluidic bioreactors can support CAR T cell activation, transduction and expansion while preserving functional activity in vitro and in vivo [169]. In the context of rapid CAR T cell manufacturing, this approach can contribute to save medium usage while dramatically reducing the required footprint to manufacture CAR T cell batches.

Microfluidic workflows integrating activation and lentiviral transduction have been shown to generate CAR T cells within 24 h while maintaining a less differentiated phenotype compared with conventional plate-based protocols. Remarkably, while the microfluidic device enabled a transduction percentage of 27%, lower transduction was observed when 48- and 6-well plates were used (resulting in 17% and 8% transduction, respectively) [170]. Besides enabling efficient oxygen and nutrient transfer, the reduced diffusion distances and high surface area to volume ratios in micro-scale channels could have potentially contributed to the improved transduction step. By favoring cell-vector contact and enhancing localized concentration of viral or non-viral vectors, microfluidic environments could offer a more efficient gene delivery, particularly under conditions of limited or no T cell activation, where maximizing gene transfer within short time windows is critical.

### in vivo CAR T cell manufacture coupled with innovative gene delivery strategies

In vivo CAR T cell manufacturing represents a paradigm shift from ex vivo cell processing toward direct genetic programming of T cells within the patient. By delivering CAR-encoding genetic material, this approach has the potential to eliminate complex manufacturing workflows, significantly shortening vein-to-vein time, besides reducing manufacturing costs and infrastructure requirements.

Early proof-of-concept studies have shown that engineered viral vectors can directly reprogram T cells in vivo. Lentiviral vectors encoding CD19 CAR have been explored to target either CD4^+^ [171] or CD8^+^ [172] T cells. Additional vector systems, including adenovirus-associated virus (AAVs), have also been explored for in vivo CAR gene delivery, demonstrating tumor regression in murine models [173].

However, viral clearance by host immune cells and the risk of untargeted delivery demand the development of efficient and safe methods to direct the delivery of the CAR-encoding genetic information to T cells. Transient CAR expression via mRNA delivery represents a fast and integration-free gene delivery approach that can generate functional CAR T cells within hours, without the risk of genomic insertion. While short-lived CAR expression may require repeated dosing or limit long-term persistence, this strategy is particularly attractive to enhance safety [174].

LNP-mediated delivery of CAR-encoding mRNA has been shown to generate potent CAR T cells with improved viability and functional persistence [15] and can be engineered for targeted delivery. LNPs can be engineered with targeting ligands (e.g., anti-CD3, anti-CD8) that preferentially deliver CAR-encoding nucleic acids to circulating T cells. Remarkably, overcoming the challenges associated with the transient expression of mRNA-engineered CAR T cells, recently, minicircle DNA encoding the CAR construct and transposase mRNA have been encapsulated in LNPs specifically targeting T cells, promoting in vivo tumor control [175]. Overall, approaches such as mRNA- or pDNA-loaded LNPs targeting CD3^+^ cells in vivo have been shown to sustain CAR expression and result in high killing efficacy of tumor cells (reviewed in [176]).

In vivo CAR T cell manufacturing could mitigate several bottlenecks inherent to ex vivo workflows. The requirements for leukapheresis, T cell activation, genetic modification, cell expansion, downstream processing including cell concentration and formulation are largely obviated, potentially enabling faster processes. Despite its promise, in vivo CAR T cell manufacturing still presents substantial scientific and regulatory challenges. Achieving sufficient and reproducible levels of CAR expression across patients remains a key hurdle, particularly due to inter-individual differences in immune status, vector biodistribution and T cell accessibility. Uncontrolled CAR expression or off-target gene delivery could exacerbate toxicities such as cytokine release syndrome (CRS) or lead to modification of non-target cell populations. In addition, the inability to apply conventional ex vivo QC assays requires rethinking potency, identity and safety assessment strategies.

### QC release

Although fast CAR T cell manufacturing protocols can accelerate patient access to autologous therapies and, in the allogeneic setting, substantially decrease CoGs and, therefore, democratize patient access to these therapies, their implementation must be paired with rapid QC assays to ensure that CAR T cell released products meet safety, identity, purity and potency requirements.

While current sterility assessment methods take usually 7–14 days to allow bacterial and fungal detection, sterility testing exploring sensitive cytometry [177] and PCR-based [178] detection methods are faster alternatives to growth-based methods and could, potentially, limit test time to few days or even hours. Strutt, et al. have shown that, using a sample volume lower than 1 mL, 16 S and 18 S amplicon sequencing coupled with ML tools can be used to detect low concentrations (≤ 10 colony-forming units (CFU) per mL, in line with Food and Drug Administration (FDA) sterility guidelines) of microbes in a turnaround time of less than 24 h [165]. Recently, a team from Seoul National University has developed a rapid sterility testing (NEST – Nanoparticle-based Enrichment and rapid Sterility Test) that uses a microfluidic chip to detect microbe concentrations as low as 1 CFU per mL within 5 to 18 h [179].

Usually, 2–3 days are required for a stable CAR expression on the cell surface and phenomena like pseudotransduction [16] can confound the reliable assessment of CAR transgene expression if evaluated prematurely. To address this, rapid workflows may rely on a combination of surrogate markers and in-process controls, such as early transgene expression kinetics or validated correlations between early molecular readouts and later CAR expression. For non-integrating or transient expression strategies, identity testing may focus on confirming delivery efficiency and expression persistence over defined short intervals.

Assays that allow for real-time monitoring of in vitro killing efficacy (e.g., impedance-based assay, such as xCELLigence Real-Time Cell Analysis technology (Agilent Technologies) and Maestro platform (Axion Biosystems), or real-time fluorescence) can provide insights into CAR T cell function. However, although real-time assays can provide faster results on the anti-tumor killing efficiency of CAR T cells, potency assays present a unique challenge in rapid CAR T cell manufacturing. Products harvested after minimal ex vivo expansion often retain a naïve or stem-like memory phenotype and may exhibit limited immediate cytotoxicity in conventional short-term killing assays. However, these products frequently demonstrate superior in vivo expansion and durable antitumor responses, highlighting a mismatch between traditional potency assays and clinical responses. Therefore, since traditional short-term cytotoxicity assays (e.g., 24–48 h killing assays) would not necessarily reflect the clinical performance of stem-like, rapidly manufactured CAR T cells whose efficacy is closely linked to their increased in vivo persistence, potency testing in rapid workflows may benefit from multiparametric approaches that include, for instance, metabolic fitness indicators, proliferative capacity upon antigen re-stimulation and their cytokine secretion kinetics, as well as on their memory/activation and exhaustion marker profiling.

It is important to note that regulatory authorities, such as FDA and European Medicines Agency (EMA), do not yet uniformly accept rapid assays as standalone real-time release criteria for cell therapy products. Instead, they are considered supportive tools that can inform a risk-based approach (introduced in Regulation 1394/2007 [180]) or conditional release strategies, provided that the assays are validated against conventional culture-based methods. Such conditional models reflect the broader regulatory trend toward flexibility for advanced therapy medicinal products (ATMPs), and rely on prior assay validation, platform consistency, in-process controls used to support timely product release, environmental monitoring and a demonstrated history of manufacturing control, to maintain a focus on ensuring product safety and sterility through validated testing strategies.

Overall, rapid CAR T cell workflows require rethinking traditional release paradigms to balance safety, regulatory compliance, and compressed timelines. A feasible minimum release panel for rapid products may include: (i) immunophenotyping (flow cytometry), (ii) viability, (iii) transduction efficiency and/or VCN and (iv) rapid sterility testing (e.g., PCR-based methods) while conventional culture-based sterility confirmation may proceed in parallel post-infusion within a defined risk-mitigation framework.

Finally, rapid manufacturing workflows often yield lower total cell numbers, limiting the amount of material available for QC testing. Therefore, careful prioritization of assays and the adoption of low-volume and high-sensitivity analytical methods are required, such as multiplexed assays, microfluidic-based analytics as well as non-destructive or inline measurements.

## Safety considerations and challenges

CAR T cell therapies can present treatment-related toxicities driven by immune cell overactivation, leading to systemic inflammatory response, a phenomenon known as CRS, or even neurological events, such as immune effector cell-associated neurotoxicity syndrome (ICANS). These adverse events arise from rapid CAR T cell expansion and activation in vivo, as well as secondary activation of endogenous immune cells, leading to the release of high levels of inflammatory cytokines such as IL-6.

Short-term CAR T cell manufacturing protocols may present added challenges by substantially limiting ex vivo culture duration. In particular, impurities such as residual lentiviral pDNA or contaminating cell populations from the original apheresis material (e.g., monocytes, tumor cells) may not be cleared during the abbreviated culture workflow. To circumvent the presence of contaminating cells in short-term protocols, an enrichment step might be required prior to T cell activation or following genetic modification, potentially offsetting gains in manufacturing speed and simplicity. Besides, rapid protocols where T cells are activated ex vivo and shortly after infused in the patient might result in products with elevated activation states at the time of administration. The presence of highly activated CAR T cells at infusion could amplify early immune activation in vivo, increasing the risk of excessive cytokine release and associated toxicities.

Several studies where rapid CAR T cell manufacturing workflows were employed have reported high-grade toxicities. Although, in these cases, CAR T cells are infused at 20–50 times lower cell doses than conventional therapies, peak blood CAR T cell levels reach similar concentrations [20], one of the factors that could lead to toxicity [181]. While rapid CAR T cell manufacturing enriches for naïve and stem-like memory phenotypes, providing superior in vivo expansion, persistence and therapeutic potency, these biologically advantageous attributes could also introduce safety risks given their rapid proliferation. Indeed, Phase I results of FasT CAR T cells infused in B-ALL patients without a prior expansion step indicated that 24% and 28% of the patients developed grade 3 or higher CRS and ICANS, respectively [10]. CAR T cells manufactured following the T-Charge rapid manufacturing protocol have also reported grade 4 CRS (6.3%) and grade 3 ICANS (12.5%) while those treated with UF-CAR also reported grade 4 ICANS [20, 38]. Potentially contributing to these observations is the fact that CAR T cells infused shortly after ex vivo activation express high levels of activation markers and may retain elevated cytokine secretion potential, potentially amplifying systemic inflammatory responses independently of total cell numbers. Although further studies would be required to unravel these mechanisms in rapid CAR T cells, Yang and colleagues, for instance, showed that, besides cell cycle-associated markers, T cell activation positively correlated with CRS severity [182].

An additional factor that may influence toxicity risk is the composition of the CAR T cell product, particularly the CD4⁺/CD8⁺ ratio. Higher proportions of CD4⁺ CAR T cells have been associated to CAR T cell treatment-related toxicity (e.g., ICANS and CRS) [183, 184] given their role in activating other immune cells involved in cytokine release and pro-inflammatory responses. In rapid manufacturing workflows, abbreviated culture durations may limit the preferential expansion of CD8⁺ T cells, therefore preserving higher CD4⁺ proportions and contrasting with conventional manufacturing, where the CD4⁺/CD8⁺ ratio typically decreases over the culture period [151]. Whether this altered balance affects the safety profile of rapid CAR T cell products remains to be determined.

To mitigate the safety risks inherent to rapid CAR T cell manufacturing, several strategies could be potentially explored, including the administration of multiple smaller infusions rather than a single dose to reduce peak systemic CAR T cell concentrations. Modulation of ex vivo activation protocols, including adjusting the duration or intensity of T cell stimulation, may help control the activation state of the infused cells, reducing the likelihood of excessive cytokine release. Importantly, allowing post-transduction periods, particularly following lentiviral integration, can minimize immune recognition of residual viral proteins (e.g., VSV-G) on the T cell surface. Besides, incorporating safety switches such as inducible suicide genes (e.g., iCasp9) [185] or targetable surface markers (e.g., truncated EGFR) [186] can allow a rapid elimination of CAR T cells in case of severe toxicity, offering an additional layer of clinical control.

Despite the promise of rapid manufacturing protocols, they also introduce complexities for QC release. The final product should be tested for sterility, mycoplasma, endotoxins and replication competent virus [187], relying on protocols that typically take days to weeks. Besides the need to develop fast QC release assays that would not delay product release into the clinic, the more limited cell yields typically obtained in short manufacturing protocols could also represent a challenge to ensure that enough cells are available for both QC assays and patient treatment. Additionally, rapid manufacturing protocols might not provide enough time for full integration of genetic cargo or lead to confounding pseudotransduction [16], therefore hindering a reliable assessment of CAR expression or VCN by flow cytometry and ddPCR (droplet digital PCR).

Importantly, shorter manufacturing timelines, resulting in cells that present a less differentiated phenotype, will likely exhibit reduced killing activity in conventional short-term cytotoxicity assays. Therefore, the adoption of fast manufacturing protocols might demand the redesign of cytotoxicity potency assays. Whereas re-stimulation assays could provide a more accurate measure of potency for products that are phenotypically immature at the time of harvest, incorporating these longer assays into the QC workflow would also extend the overall release timeline.

Finally, in vivo CAR T cell generation would imply a shift from in vitro potency assessment to real-time in vivo monitoring. Increased emphasis will likely be placed on vector characterization, biodistribution, targeting specificity and post-infusion monitoring. Ensuring controlled and selective gene delivery becomes critical to minimize off-target modification and uncontrolled immune activation.

Given the advancement of the field towards rapid manufacturing protocols and the evidenced efficacy of these CAR T cell products at lower cell doses, assays designed to evaluate the safety of the manufactured CAR T cells should be re-evaluated regarding both cell efficacy and toxicity. Developing mechanism-informed and risk-based safety assessment strategies will be essential to enable broader adoption of accelerated CAR T cell manufacturing workflows.

## Conclusion

Rapid CAR T cell manufacturing represents a fundamental shift in cell therapy bioprocessing, moving away from prolonged ex vivo expansion and maximized cell yields towards time-compressed workflows that, at the expense of high CAR T cell doses, prioritize cellular fitness, enrichment of naïve and stem-like memory CAR T cell phenotypes and, ultimately, in vivo functionality and persistence.

The success of rapid manufacturing workflows is governed by the modulation of several process parameters, including the dynamics of T cell activation, exposure to the vector carrying the DNA- or mRNA-encoded CAR information, cytokine composition and oxygen availability, for instance. Early process decisions can strongly influence the long-term in vivo response and should therefore be carefully considered when designing CAR T cell bioprocesses.

Technological enablers, including closed and automated platforms, microfluidic culture systems, real-time PAT tools and AI-enabled predictive models, will be essential not only to improve reproducibility, but also to enable dynamic bioprocess interventions within narrow temporal windows. Besides, in rapid CAR T cell manufacturing paradigms, QC release must be adapted to operate under three defining constraints: severely limited time for testing, reduced cell yields available for analysis, and products that are phenotypically immature at the time of harvest. Addressing these constraints requires a shift from endpoint, growth-based assays toward rapid and sensitive methods that can support timely and robust product release.

Aligning QC release with the biological and process realities of rapid CAR T cell manufacturing will be critical to fully realizing the clinical and economic potential of these accelerated therapies. As regulatory frameworks evolve to accommodate innovative manufacturing paradigms, early engagement with regulatory authorities will be essential to define acceptable surrogate assays, conditional release strategies and post-infusion monitoring requirements.

Together, these efforts can support the development of faster, cost-effective and decentralized CAR T cell therapies, expanding access to a broader patient population while preserving, or even enhancing, their therapeutic efficacy. Overall, rapid workflows may not only improve existing CAR T cell therapies but also reshape how cellular (immune)therapies are engineered and delivered to the patient.

## Data Availability

No datasets were generated or analysed during the current study.

## Abbreviations

AAV: Adeno-associated Virus
AI: Artificial Intelligence
ALL: Acute Lymphoblastic Leukemia
AML: Acute Myeloid Leukemia
BCMA: B-cell maturation antigen
CAR: Chimeric Antigen Receptor
CFU: Colony-Forming Unit
CLL: Chronic Lymphocytic Leukemia
CMML: Chronic Myelomonocytic Leukemia
CoG: Cost of Goods
CR: Complete Response
CRS: Cytokine Release Syndrome
ddPCR: Droplet Digital PCR
DO: Dissolved Oxygen
FAO: Fatty Acid Oxidation
FDA: Food and Drug Administration
IFN: Interferon
LDL-R: Low-Density Lipoprotein Receptor
LNP: Lipid Nanoparticle
MDS: Myelodysplastic Syndrome
ML: Machine Learning
MM: Multiple Myeloma
MOI: Multiplicity of Infection
NDMM: Newly-Diagnosed Multiple Myeloma
NHL: Non-Hodgkin’s Lymphoma
ORR: Overall Response Rates
OXPHOS: Oxidative Phosphorylation
PAT: Process Analytical Technology
PBMC: Peripheral Blood Mononuclear Cells
PoC: Point-of-Care
QC: Quality Control
ROS: Reactive Oxygen Species
R/R: refractory/relapsed
RT: Richter’s Transformation
SCT: Stem Cell Transplant
TCR: T Cell Receptor
TNF: Tumor Necrosis Factor
VCN: Vector Copy Number
VSV-G: Vesicular Stomatitis Virus G protein
